# Utilization of 3D printing technology to facilitate and standardize soft tissue testing

**DOI:** 10.1038/s41598-018-29583-4

**Published:** 2018-07-27

**Authors:** Mario Scholze, Aqeeda Singh, Pamela F. Lozano, Benjamin Ondruschka, Maziar Ramezani, Michael Werner, Niels Hammer

**Affiliations:** 1 Department of Anatomy, University of Otago, New Zealand Department of Anatomy, Dunedin, New Zealand; 2 Institute of Materials Science and Engineering, Chemnitz University of Technology, Chemnitz, Germany; 3 Institute of Legal Medicine, Medical Faculty University of Leipzig, Leipzig, Germany; 4 Department of Mechanical Engineering, Auckland University of Technology, Auckland, New Zealand; 5 Department of Trauma, Orthopedic and Plastic Surgery, University Hospital of Leipzig, Leipzig, Germany; 6 Fraunhofer Institute for Machine Tools and Forming Technology, Dresden, Germany

## Abstract

Three-dimensional (3D) printing has become broadly available and can be utilized to customize clamping mechanisms in biomechanical experiments. This report will describe our experience using 3D printed clamps to mount soft tissues from different anatomical regions. The feasibility and potential limitations of the technology will be discussed. Tissues were sourced in a fresh condition, including human skin, ligaments and tendons. Standardized clamps and fixtures were 3D printed and used to mount specimens. In *quasi-static* tensile tests combined with digital image correlation and *fatigue* trials we characterized the applicability of the clamping technique. Scanning electron microscopy was utilized to evaluate the specimens to assess the integrity of the extracellular matrix following the mechanical tests. 3D printed clamps showed no signs of clamping-related failure during the *quasi-static* tests, and intact extracellular matrix was found in the clamping area, at the transition clamping area and the central area from where the strain data was obtained. In the *fatigue* tests, material slippage was low, allowing for cyclic tests beyond 10^5^ cycles. Comparison to other clamping techniques yields that 3D printed clamps ease and expedite specimen handling, are highly adaptable to specimen geometries and ideal for high-standardization and high-throughput experiments in soft tissue biomechanics.

## Introduction

Standardization of mechanical experiments involving biological soft tissues when tested under strain remains an ongoing issue with negative impact on the accuracy and validity. Soft tissues are characterized by a number of special characteristics including anisotropy, heterogeneity, viscoelasticity and inter-individual variation. These variables are further potentiated particularly in human samples by the post-mortem delay and therefore autolysis, alterations in water content, and underlying traumatic or internal pathology, complicating the standardization of the mechanical tests. Consequently, to obtain robust results, large sample sizes are necessary to outweigh the statistical scatter which is likely to occur.

To overcome issues such as material slippage in uniaxial tensile tests, a number of adjustments and fixtures have been introduced^1–3^, including steel clamps with roughed surface areas^4,5^ and high friction areas^6–9^, cryogenic clamps^10,11^ or pneumatic clamps^1–3,9,12–14^. However, these existing methods have limitations regarding their applicability to the soft tissues, especially regarding the formation damage due to avulsion at the clamping site or the risk of temperature-induced changes in the mechanical behavior. In recent years, we have developed a technique called partial plastination to address material slippage^15–17^. Partial plastination helps to minimize material slippage by using ceramic-reinforced polyurethane resins at the mounting sites of the tissue and leaving behind an area of native tissue for mechanical testing with a sharp boundary to the plastinated area^15,16^. This technique however comes with certain limitations including the time-consuming preparation, the need for highly specialized plastination equipment such as vacuum pumps and casting fixtures, and the use of further chemicals such as acetone with unknown effects on tissue mechanics and potential hazard for the users. Furthermore, the difficulty of positioning soft tissues in a vertical test setup (involving the effects of gravity) can introduce errors during the clamping by deviations from the axial position which could also lead to variances in strain and force recordings.

As a consequence, we aimed to explore alternative techniques which may facilitate tissue clamping, and aid in standardizing the clamping of soft tissues for biomechanical testing in a less time-consuming manner. 3D printing has meanwhile become broadly available, and such professional extrusion solutions can be utilized for customizing and printing fixtures and adjustments for biomechanical testing using commercially-available filaments. Furthermore, it can be utilized to provide affordable add-ons to existing testing devices all over the world, going beyond just soft-tissue biomechanics. The possibility of sharing existing digital models enables a broad availability and exchange of research and knowledge. 3D printing may also be used for clamping mechanisms, and variations in clamping design appear to be eased by the rapid-prototyping approach with the ubiquitously-available software.

This report will describe our preliminary experience using 3D printed clamps to mount human soft tissues from three different anatomical regions with different properties, including skin, ligaments, and tendons, and compare this method to our previous experience with partial plastination^15,17–20^. The feasibility and potential limitations of the technology will be discussed, and two scenarios will be shown for *quasi-static* and *cyclic (fatigue)* tests.

## Materials and Methods

**Figure 1:**
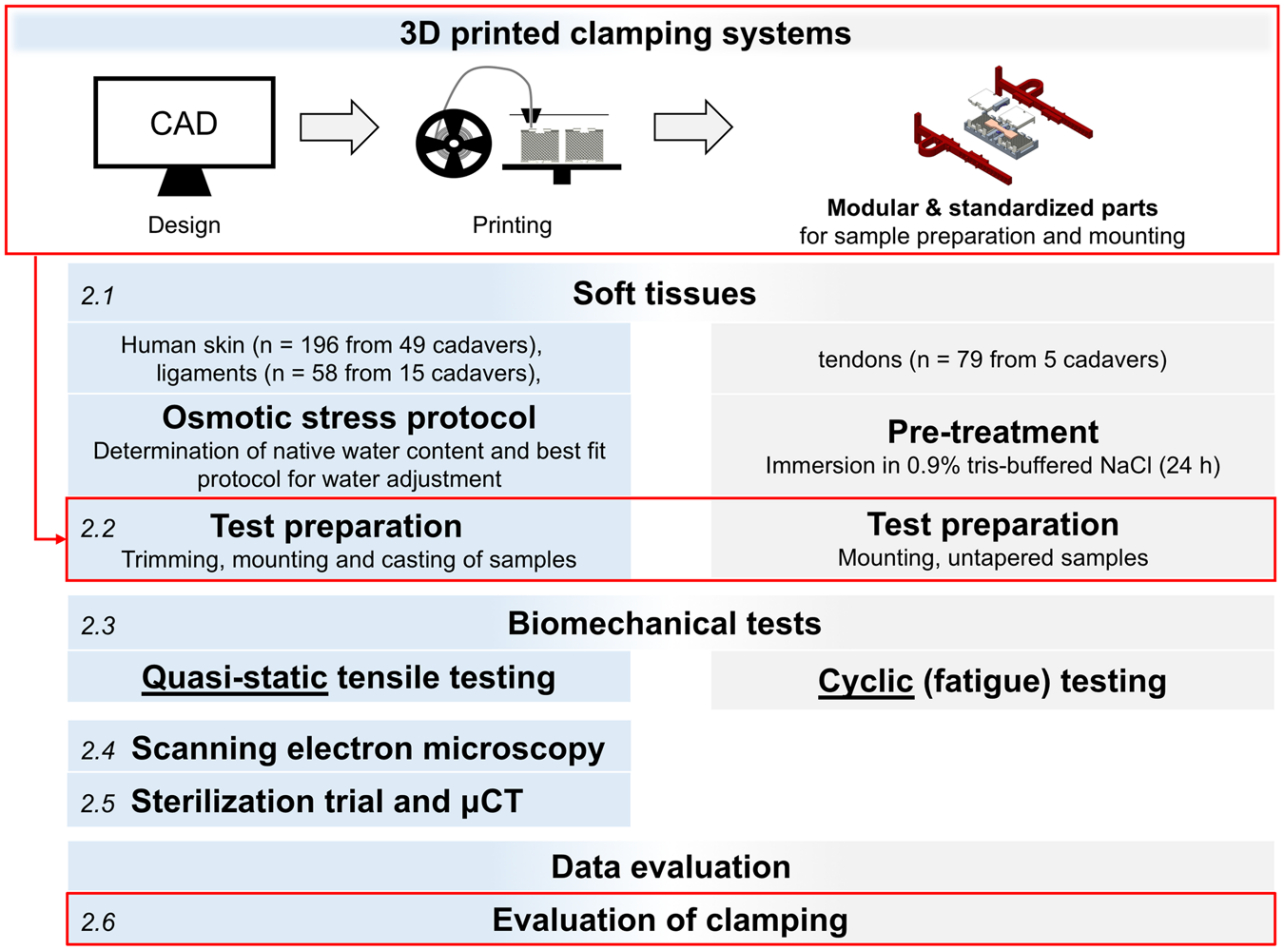
Standardization in material testing and test setup. Focus of this study will be the boxes highlighted in red. The figure is numbered in italics according to the steps described in Materials and Methods.

A general overview of the workflow for the experiments and evaluation of this study is given in Fig. 1. All experiments were performed in accordance with relevant guidelines and regulations.

### Tissue sampling and water content adaptation

Human tissues were sourced from different anatomical regions for these experiments in a fresh and unfixed condition. Scalp skin samples (n = 196 from 49 cadavers, age range 6–94 years), and iliotibial band tissues (ligaments; n = 58 from 15 cadavers, age range 6–97 years) were obtained from human cadavers in forensic medicine (ethical approvals 486/16 EK and 156-10-12072010 from the University of Leipzig, Germany). Flexor digitorum superficialis and profundus tendons (n = 79, age range 77–91 years) were harvested from the arms of another five human cadavers (ethical approval H17/20 from the University of Otago, New Zealand). The different age range of the tissues is explained by the different cohorts in anatomy environments as opposed to forensic medicine. A protocol was applied to adapt the tissues’ water content. For human skin and ligament samples, the water content was adapted to the average of the native condition in the sample population^20,21^. These samples were later tested in *quasistatic* tensile tests with a short test duration (1–2 min), where the adaptation minimized the influences related to age and the post-mortem interval. Human tendons were pre-treated in 0.9% tris-buffered saline, because of the long test duration (up to 12 hours) and the possibility to moisten the samples during the tests in predefined intervals with the same liquid (every 20 minutes).

### 3D printed clamping systems for preparation and mounting

The clamping systems, including clamps and fixtures were developed by computer-aided design (CAD) in Creo 4.0 (PTC software, Needham, MA, USA). 3D printing was conducted using the Ultimaker 3 Extended (Ultimaker B.V., Geldermalsen, The Netherlands), applying the Fused Deposition Modelling (FDM) technique. All models were processed with the Ultimaker Cura 3 slicing application prior to printing. Commercially-available filaments of acrylonitrile butadiene styrene (ABS), nylon (polyamide), polylactic acid (PLA) and thermoplastic polyurethane (TPU) were trialed at printing parameters according to the distributor (Ultimaker B.V.). For all 3D prints, the parts were produced with 100-micron layers using a 0.4-mm brass nozzle. The clamps were printed in an upright position for a lateral orientation of the pyramids, allowing a very detailed quality of these surface structures.

#### Tables and templates for standardizing tissue dimensions

In the first step, specimen tables suitable for tissue preparation prior to testing were designed to include cutting into a ‘dog-bone’ shape and casting (determination of the cross-sectional area). The tables were constructed so that different sample dimensions could be mounted, varying between 5.0 and 80.0 mm to account for different tissue geometries and dimensions. The tables in the final design introduced here primarily have the function of simplifying tissue preparation and offer an approach to standardize the dimension and geometry of soft biological samples. They were printed of PLA and consisted of the following setup and parts:

- Base with a central area to place the parallel testing area of the sample, one central and two peripheral openings. The central opening serves for determining the samples’ cross section, the peripheral opening for the clamps.
- Cutting template, according to the ISO 527-2 (International Standard Organization, 1996) to cut samples into a ‘dog bone’ shape.
- Molding device: These clips were used as micro versions of impression trays commonly used in dentistry. Clips were attached to the devices, to align them in parallel and to minimize the use of impression material required for the cast (determination of specimen cross section). The resulting impressions served to determine the samples’ cross-sectional areas.

**Figure 2:**
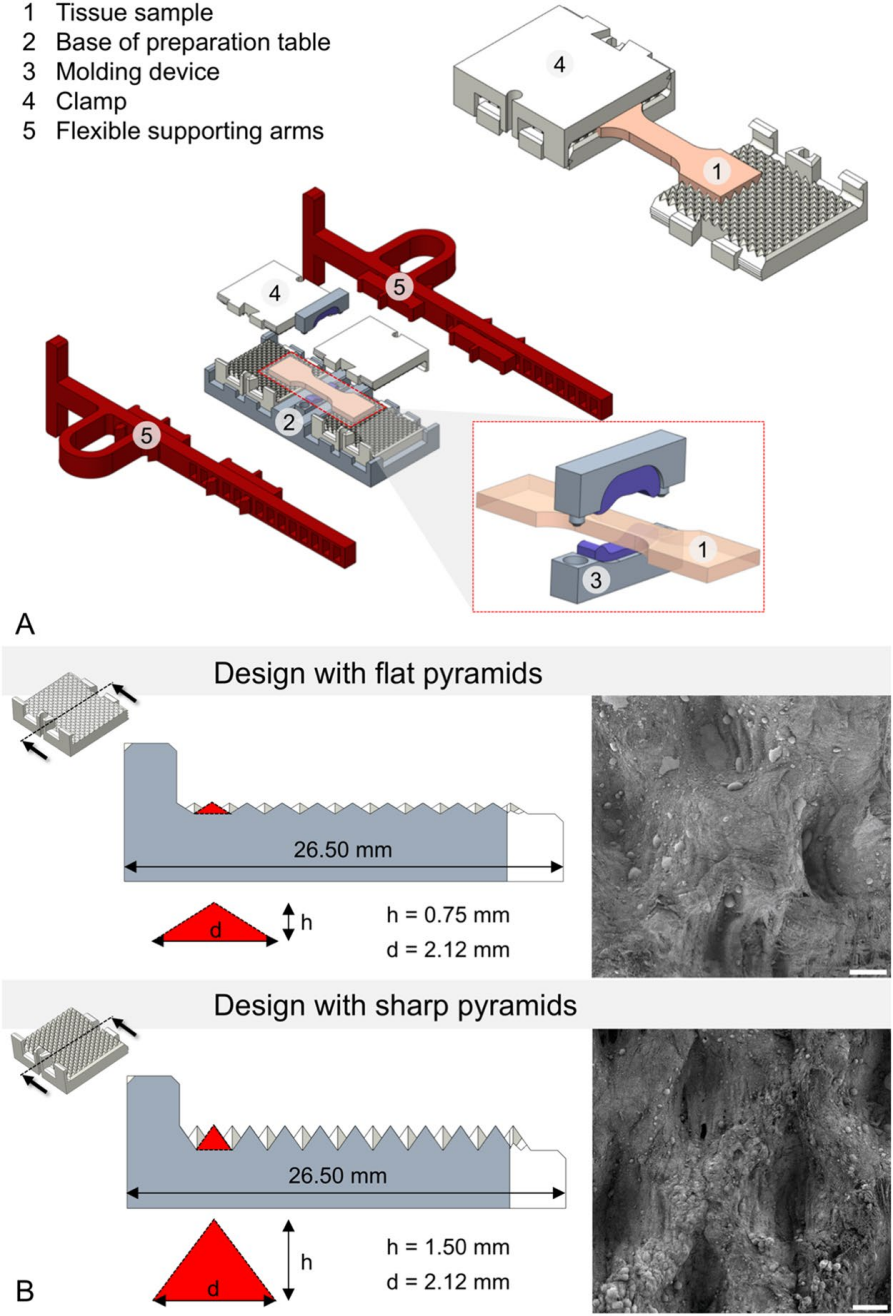
(**A**) Mounted sample in the self-locking clamps (top right), and schematic representation of the preparation table (center of image) including a (enlarged) molding tool for the determination of cross sections of the samples prior to testing (right bottom). (**B**) Clamp designs with 4-sided pyramid structures in cross-sectional view. Pyramid dimensions (d = diagonal, h = height) are enlarged. Additionally, corresponding scanning electron microscopy images of clamped ligament tissues after testing at 25x magnification are represented at the right. Note the elongated impressions in the ligament tissues clamped with the sharp pyramids (scale bar 100 μm).

**Figure 3:**
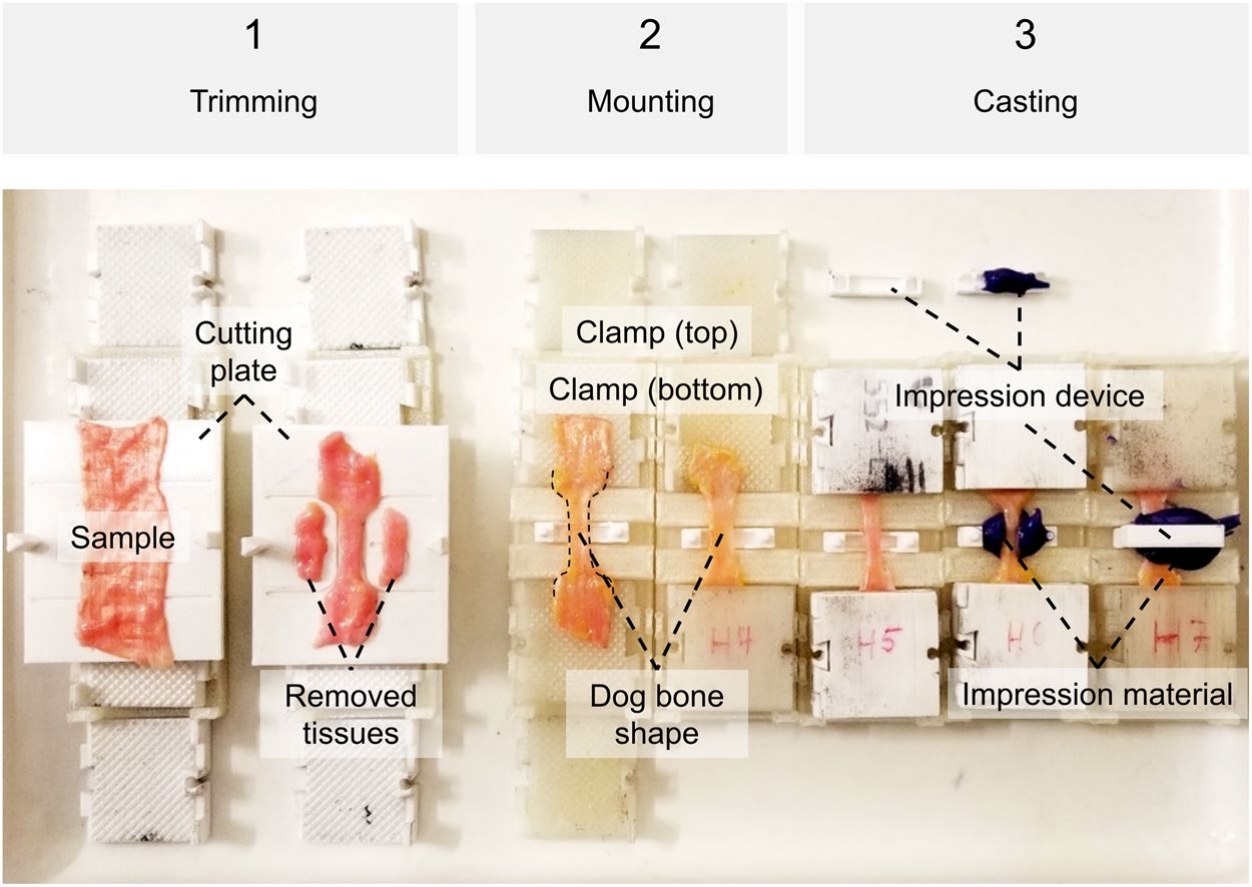
Steps of trimming, mounting and casting specimens. (**1**) The cutting template consists of a bottom and a top plate (not shown), and a guide rail allowing for exact positioning. Radii and lengths can be adjusted according to the ISO standard to get a ‘dog bone’ shape. Removed tissues can be used for further analyses, e.g. on ultrastructure or water content. (**2**) Each clamp consists of four identical parts interlocking pairwise, mounted to the end of the specimen pairwise in a fixed sample length given by the mounting Table. (**3**) Casts of the specimens’ cross-sectional areas can optionally be made using a designated opening at the mounting table. Impression material may either be directly placed in this opening, or in a two-part impression device shown in the picture, allowing for less use of the impression material.

Figure 2A represents an exploded assembly drawing of the complete preparation table with a tissue sample and molding devices as well as the flexible supporting arms. The stepwise preparation of tissues with this approach is shown in Fig. 3.

#### Clamping design and supporting arms for tissue mounting

Clamping devices of varying geometry and surface characteristics were trialed using different materials. The clamping area itself was built to have included pyramid structures on both sides, which interlock with the sample by form and force closure when being compressed. For the reusable design, the walls were constructed to interlock and to have a self-locking mechanism (2-part design, Fig. 2A). Additionally, a breakable single-use design based on the same structures and clamping mechanisms was made especially for small clamp opening widths, where the use of the 2-part design is impossible. These clamps were designed as tubes with two openings as 1-part design and later refined especially for *fatigue* testing purposes, given that such machines have much smaller clamping widths, only allowing for relatively small mounts. Preliminary experiments were conducted for both sharp- and smooth-edged pyramid tips (Fig. 2B). Sharp pyramids were found out to be more suitable for *quasi-static* experiments and/or when testing thicker samples, where a greater interlock with the samples is necessary, therefore forming the basis for the application with full thickness skin samples. Smooth pyramids were found out in preliminary tests to offer suitable interlock with thinner samples like tendons or ligaments. They were especially used in the breakable design for *fatigue* testing of the tendons. As part of this design, flexible supporting arms were constructed out of TPU to facilitate the mounting of the tissues in the material testing machine at a standardized clamping length. Prior to the tests, the clamps were soaked with isotonic saline to circumvent dehydrating effects of the tissue samples from the dry plastics.

### Biomechanical tests

Tensile mechanical experiments were conducted with all tissues at 21° Celsius and following osmotic adaptation of the water content (skin and ligaments) or moistening (tendons), according to the flow chart given in Fig. 1.

**Figure 4:**
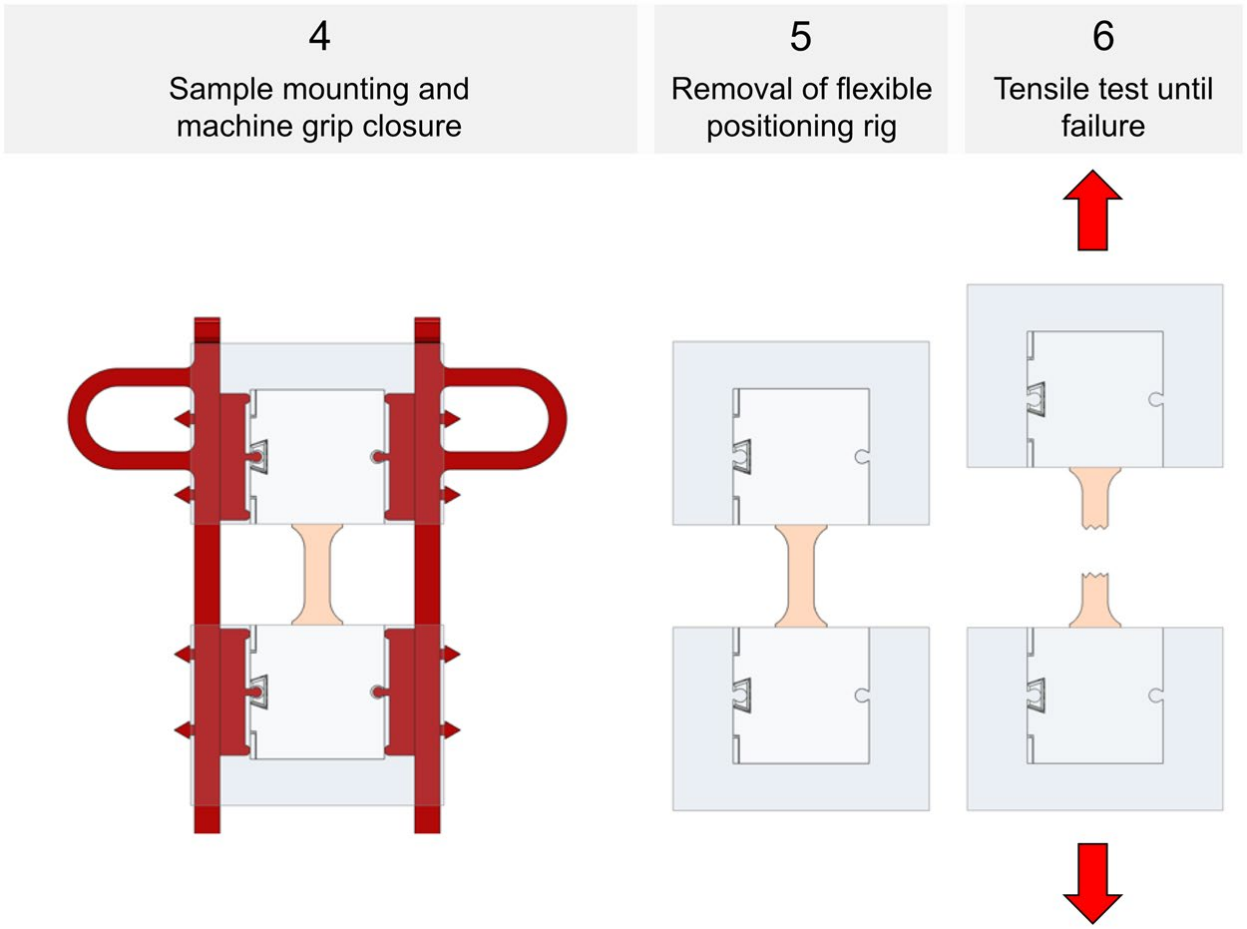
Specimen mounting and removal of positioning ring for *quasi-static* experiments.

**Figure 5:**
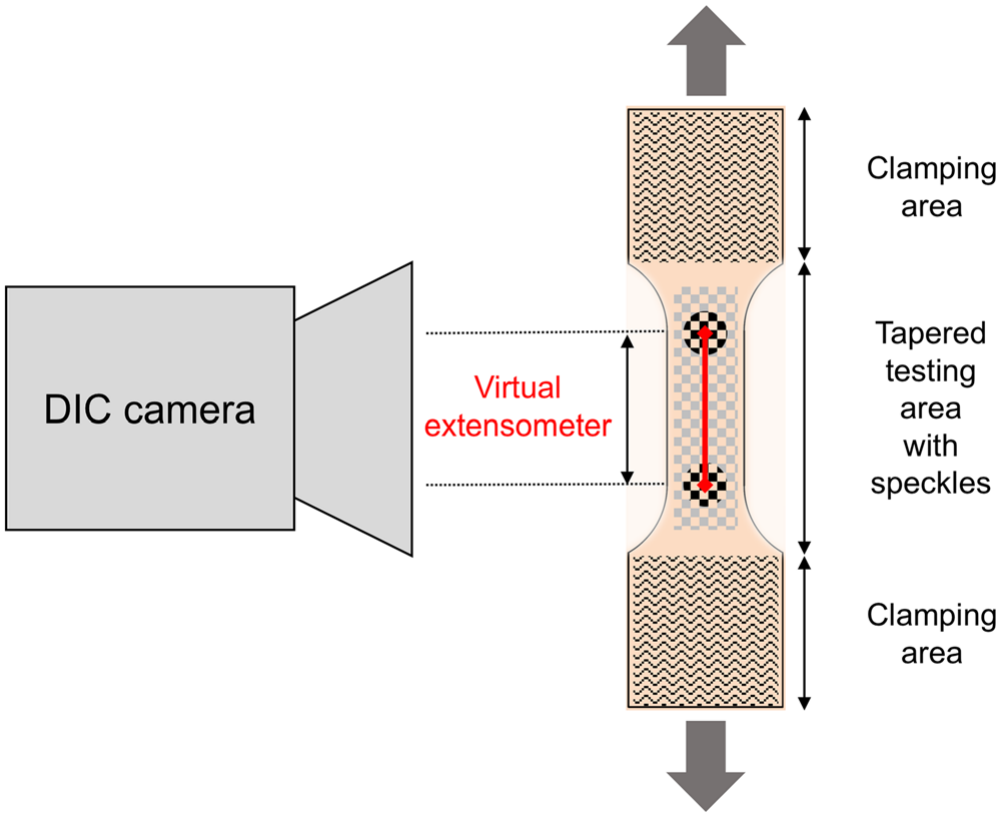
Principle of digital image correlation used for the strain evaluation in the *quasi-static* experiments: A calibrated camera recorded the tapered testing area of the samples during tensile deformation. By the evaluation of randomized speckles at the surface, the system can track facets and calculate an in-plane surface strain field. The nominal strain for the evaluation of the tests was further calculated by a perpendicular virtual extensometer between two points defined in the parallel testing length of each sample.

Following the tapering, mounting and clamping as shown in Figs 3 and 4, *quasi-static* experiments were conducted for the skin and ligament samples in a universal testing machine (Zwick Roell 20 kN Allround Table Top Z020 and 2.5-kN Xforce P load cell, Ulm, Germany). A pair of pneumatic grips Zwick type 8297 was used to mount the 3D printed clamps in the machine and to facilitate a constant compressive gripping force (6 bar operative pressure which equals 6500 N gripping force and 10 MPa pressure at the 3D printed clamps). The samples tested *quasi-static* were preconditioned to 20 load-unload cycles conducted at 30% of the assumed maximum stress before the tissues underwent a final cycle reaching until material failure, indicated by a >30%-decrease from the previous maximum force. Crosshead displacement was 20 mm/min and sampling rate was 100 Hz. A strain measurement by contacted extensometers was impossible when testing soft tissues given the high risk of sample damage. Therefore, a contactless optical (digital) image correlation system (DIC; Q400, Limess, Krefeld, Germany and Istra4D, Dantec Dynamics, Skovlunde, Denmark) was synchronized with the testing device for strain measurements (image sampling rate 10 Hz). This technique offers a precise evaluation of strain directly at the surface of the sample during deformation by tracking of speckles having a stochastic pattern with high contrast. A schematic overview of this approach is given in Fig. 5.

**Figure 6:**
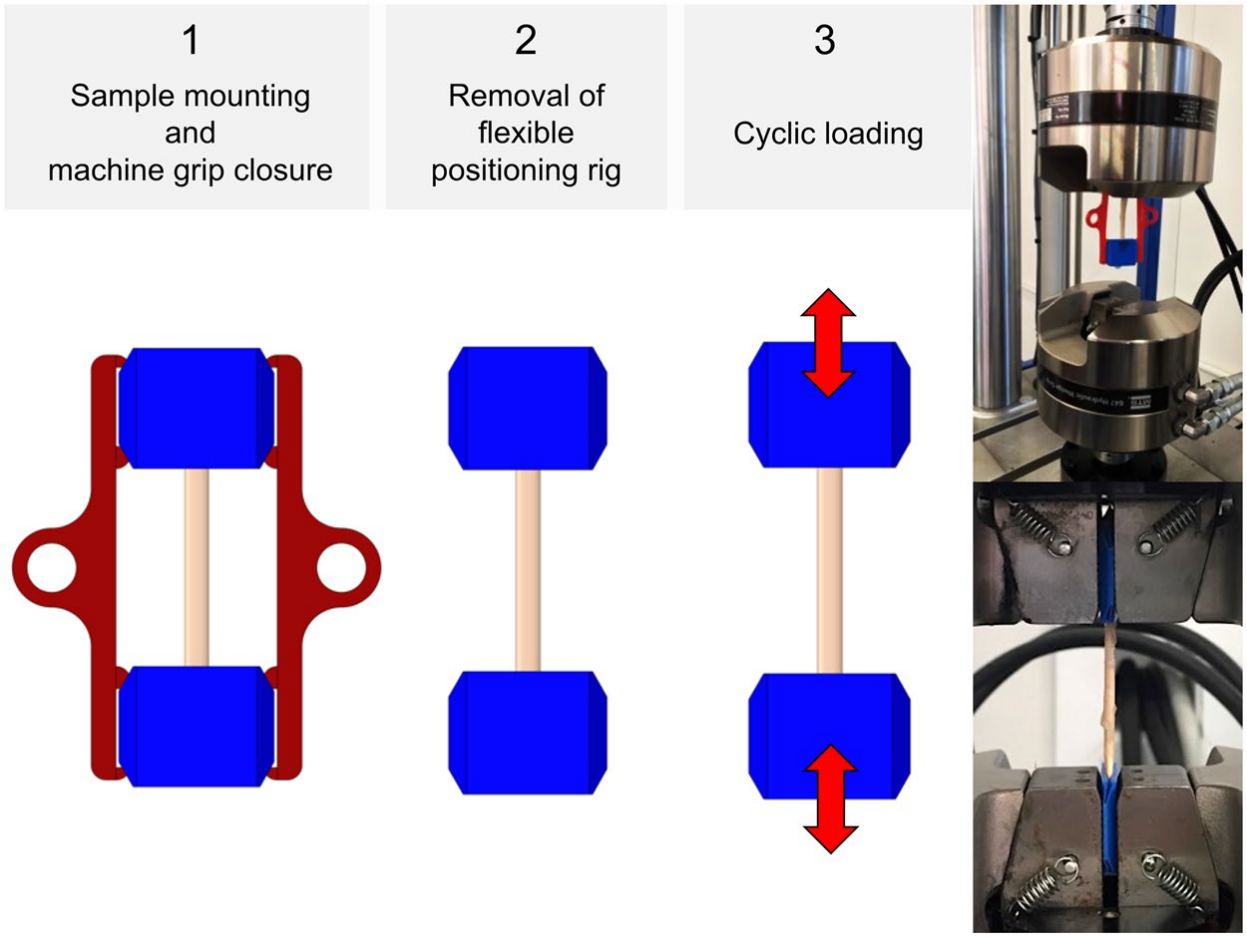
Specimen mounting for *cyclic* tests (left and middle), and photographs showing *fatigue* experiments of human flexor tendons.

The untapered flexor tendons underwent cyclic *fatigue* testing using an MTS Landmark Servohydraulic Test System (Eden Prairie, MN, USA) with hydraulic grips. The samples were mounted in a similar manner at a slightly lower clamp pressure than in *quasi-static* tests (6 MPa) to avoid clamping related micro-damages and resulting issues in *fatigue* tests. Following a preload of 10 N, *cyclic* loading was conducted with a minimum load of 10 N and a maximum load ranging from 200 N to 500 N. Mounting of the samples was conducted as shown in Fig. 6. The tests were effectuated at a frequency of 4 Hz, and the samples remained in tension throughout the tests. For all experiments, the type and location of failure of the biological tissues were recorded. Additionally, the samples were inspected for material slippage and for potential failure of the clamps after testing.

### Scanning electron microscopy

Following the experiments, scanning electron microscope analyses of representative samples were conducted using a JSM-6700F field emission microscope (JOEL Ltd., Tokyo, Japan) at magnifications between 25x and 15,000x to examine the specimens’ failure sites. Random samples were chosen of the skin (n = 48 regional samples) and ligaments (n = 12 regional samples).

### Micro computed tomography (μCT) and sterilization

Six clamps were scanned using a General Electric phoenix v/tome/x s μCT (GE Measurement & Control Solutions, Wunstorf, Germany) with an isovoxel size of 110 µm prior to and following steam sterilization. Three clamps underwent sterilization at 121 °C for 20 minutes at 2.048 bar with a drying time of 10 minutes. The remaining three clamps were steam sterilized at 134 °C for 10 minutes at 3.043 bar, followed by a drying time of 20 minutes. The resulting geometries were then assessed using VGSTUDIO MAX 3.1 (Volume Graphics GmbH, Heidelberg, Germany).

### Statistical evaluation

All statistical comparisons were done using Microsoft Excel (Microsoft Corp., Redmond, WA, USA), SPSS version 24 (IBM, Armonk, VA, USA) and Graphpad Prism 7a (GraphPad Software Inc., La Jolla, CA, USA). Cramér’s φ correlations were determined following the Chi-square test to assess the association between extracellular matrix integrity and the location site of investigation as an indicator of protein preservation. This was done at the clamping site, the border between the clamping and the testing area, and the failure site. *P* values of ≤ 0.05 were considered as statistically significant.

## Results

### Applicability and first experiences with the technique

The combination of tissue preparation, clamping and mounting with 3D printed parts and accessories allowed for soft tissues to be tested in a highly-standardized and high-throughput approach. PLA was primarily used for the tables, templates and clamps, and TPU for the specimens´ mounting frame, which consisted of two supporting arms. With the given design, the arms remained at the samples’ clamps when positioned in the testing machine and this led to the samples being compressed between the closing grips. Consequently, the supporting arms needed to be smaller than the gripping width and elastic enough to be pulled out of the fully compressed clamps and inserted without excessive wear of breakage, allowing for reuse. The framed design helped to maintain the sample at a parallel clamping position and at a predefined clamping length without alterations related to manual handling of the sample itself. Moreover, it formed one of the prerequisites for rapid sequence/high-throughput testing of samples at high standardization.

**Figure 7:**
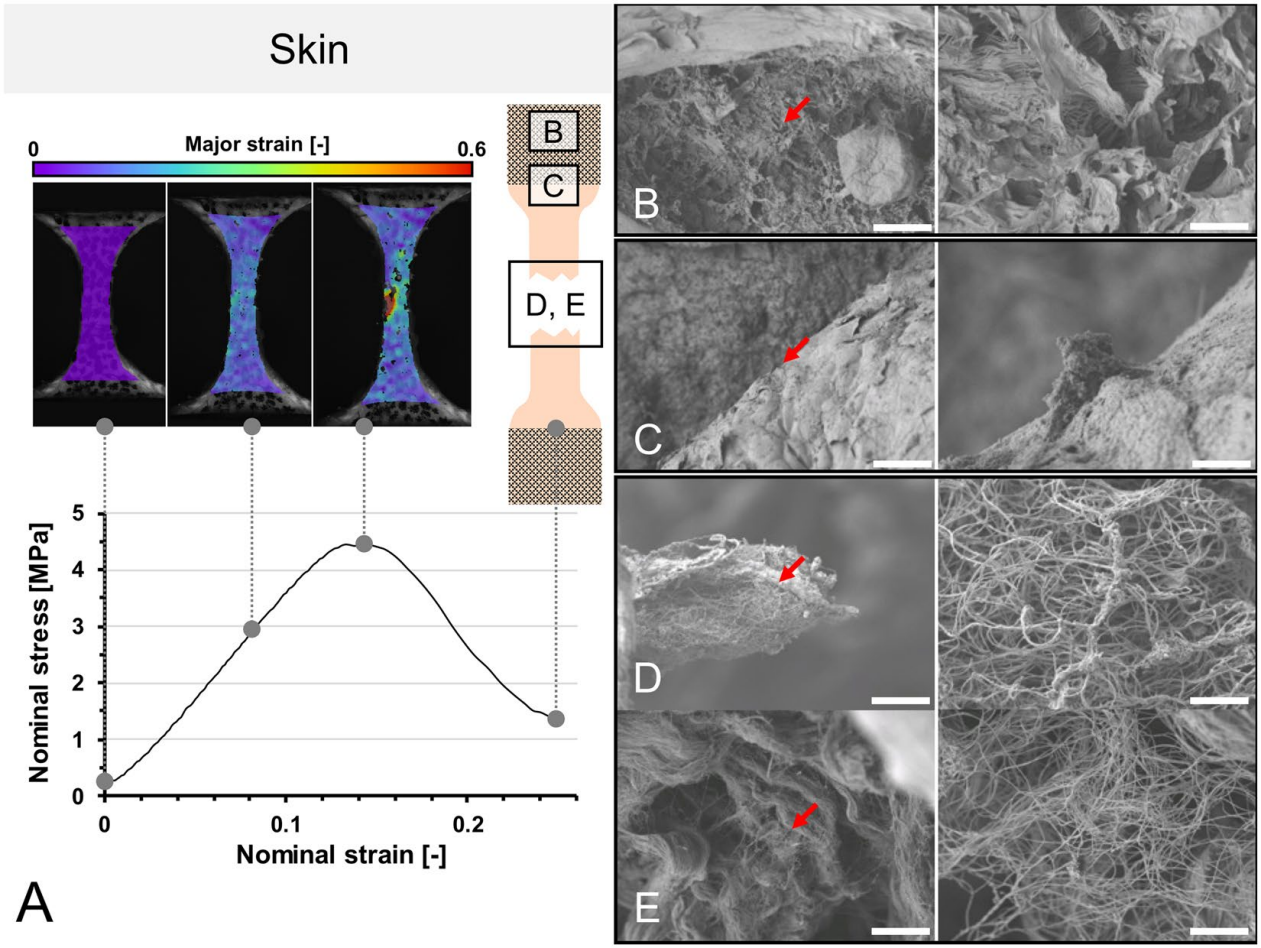
(**A**) Stress-strain curve of human calvarian skin and the corresponding strain fields from digital image correlation in three steps before the specimen fails. A schematic sample with squares indicates the area where tissues were removed for electron microcopy. (**B**–**E**) Scanning electron microscopy of human calvarian skin. Left side 500x magnification (scale bar 40 μm), right side 5,000x magnification (scale bar 4 μm), red arrows indicate the area of magnification for the right picture. (**B**) Clamping area. Images correspond to skin obtained from the upper parietal bone area of an infant male. Partially ruptured keratin sheets in low magnification. Partial collagen fiber rupture can furthermore be seen in higher magnifications. (**C**) Edge area. Images correspond to skin obtained from the upper occipital bone area of a geriatric male. Partially ruptured keratins can be seen in low magnification. In higher magnifications, ruptured collagen bundles are found surrounded by keratin sheets. (**D**) Failure area. Images obtained from upper parietal bone area of a geriatric male. In this area, mainly collagen bundles were found, with both retracted and intact areas, building a dense network. (**E**) Failure area. Obtained from the area upper superficial to the occipital bone of an infant male. At lower magnifications, a similar pattern can be seen as in the geriatric sample. At higher magnifications, it can be seen that a similar area is composed of lesser collagen at a lower density but higher quantity, which may be partially responsible for higher elasticity and lower ultimate stress features.

**Figure 8:**
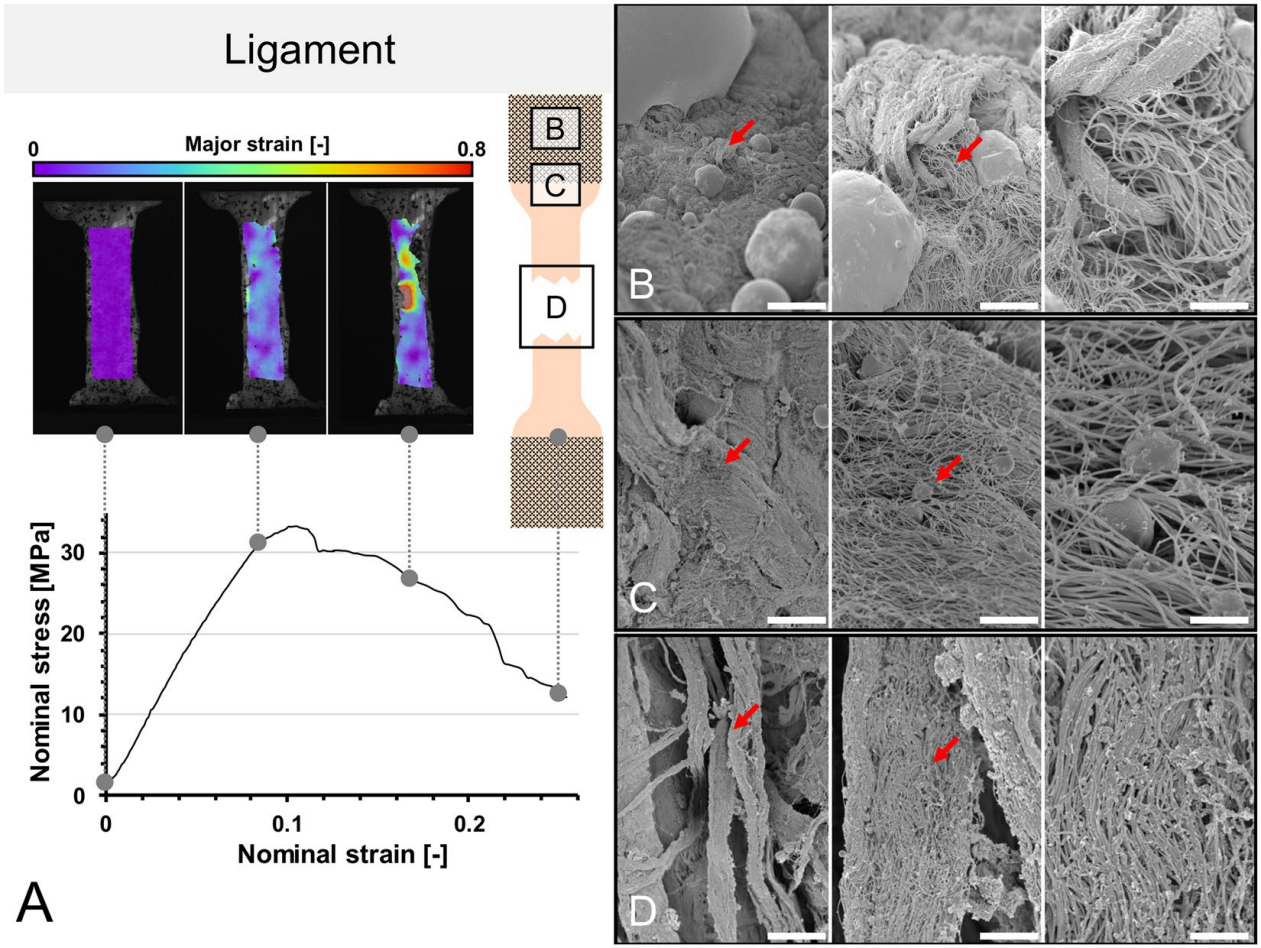
(**A**) Stress-strain curve of human ligament and the corresponding strain fields from digital image correlation in three steps before the specimen fails. A schematic sample with squares indicates the area where tissues were removed for electron microcopy. (**B**–**D**) Scanning electron microscopy of ligament samples. Left side 1,000x magnification (scale bar 20 μm), middle 5,000x magnification (scale bar 2.5 μm), right side 15,000x magnification (scale bar 1 μm), red arrows indicate the area of magnification for the next picture. (**B**) Clamping area. Images correspond to the area under the pyramids of the clamps. Collagen fibers remained partially to fully intact in all magnifications. (**C**) Edge area. Intact and partially ruptured collagen fibers are seen, indicated by disruption of fibers and ball-shaped collagens in higher magnifications. (**D**) Failure area. Lumpy collagen appearance, collagen disarrangement and collagen balls indicate multiple sites of rupturing in the failure areas.

**Figure 9:**
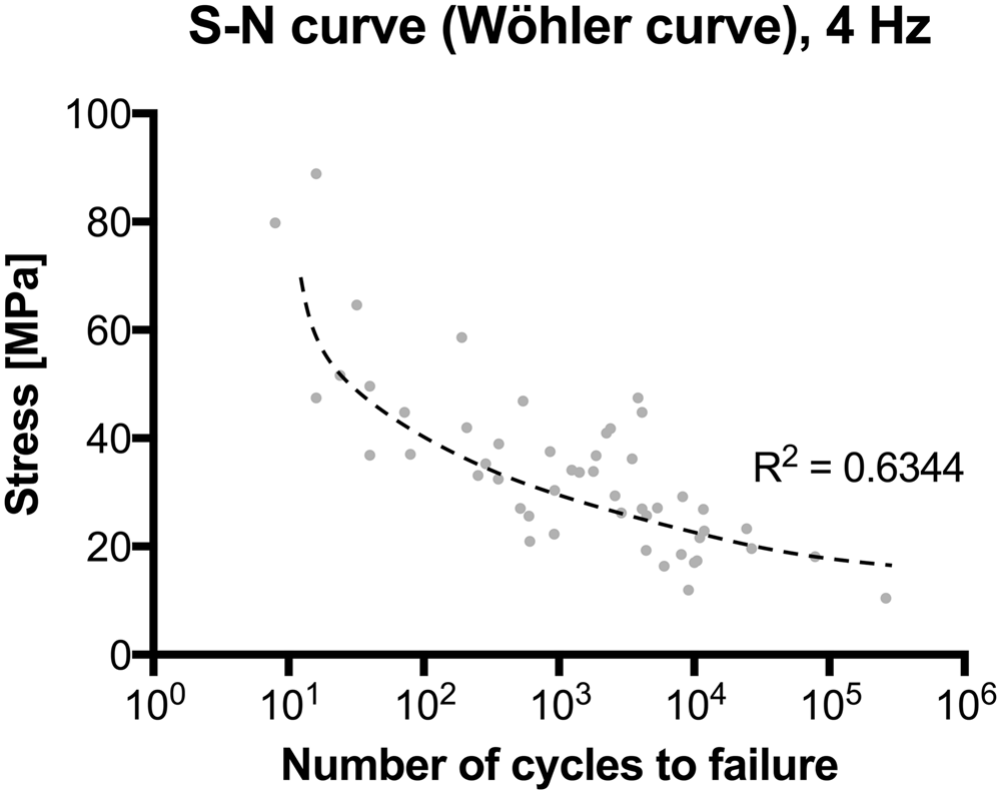
Stress-cycle (S-N) curve (Wöhler curve) for human flexor tendon *fatigue* behavior tests.

In pilot trials, a sample throughput of up to 32 samples/day (4 specimens/hour) was achieved for the *quasi-static* uniaxial tensile tests with combined DIC, beginning with the freshly-harvested and untapered sample. Examples of load-deformation curves and the resulting load-deformation appearance of tissue under scanning electron microscopy (SEM) are given in Figs 7 and 8. In case of the *fatigue* testing setup, cycles beyond 1.6 × 10^5^ were reached, allowing to obtain *fatigue* cycle stress-cycle (S-N) characteristics (Wöhler curves) for 50 flexor tendons (the remaining 29 samples were used for pilot trials). Depending on the used force, the testing time reached intervals up to 11 hours. A scatter plot comparing the number of cycles to failure and used stress is given in Fig. 9. Sharp pyramid geometries in the clamping areas gave more accurate results in the higher strains of the *quasi-static* material tests reaching to material failure, whereas flatter pyramid geometries were more useful for the *fatigue* tests, allowing for higher cycles before material failure occurred. Clamps with sharp pyramids (four per specimen, two interlocking per side, see Fig. 2) were primarily used for thicker samples and static tests, which involved the skin specimens. Clamps with flatter pyramids (2 per specimen, one per side) were primarily used for the fatigue tests and thinner samples. A total of 200 clamps with sharp pyramids were used for the skin tests, and another batch of 60 clamps with flat pyramids for the ligament tests, resulting in an average reusability of 4 times for both designs, ranging between 3x and 5x reuse.

In contrast, the partial plastination technique requires a two-day setting^17–21^. Preparing the samples to the step of the embedding basically follows the same steps as above, succeeded by surface coating of the central specimen part to be evaluated with gelatin for protection purposes and mounting before the samples’ ends are dehydrated in acetone^15^. In the following step using acetone, the specimens were prepared for embedding in the ceramic-polyurethane resin, ideally applying a mild vacuum for forced impregnation. Subsequently following the curing, the removal of the templates, protruding resin and of the gelatin covering the testing area of the samples was necessary and time-consuming. The steps and chemicals used makes the entire process necessary to adjust the water content following the plastination. Preparation time averages 20–30 minutes per sample, and a full two-day time frame is necessary to test specimens in a standardized manner, resulting in a potential throughput of 16 samples in 2 days (1 sample per hour), beginning with a freshly-harvested sample. Sand paper clamping at first step appears to be easier to carry out but brings significant issues with slippage or avulsion and lacks in opportunities for standardization.

### Material failure, slippage and clamping failure

Using the approach with 3D printed accessories, failure occurred in the central area in 189/196 human skin samples (96%) in the *quasi-static* experiments and in 48/58 of the ligament samples (83%). In case of the *fatigue* tests with the untapered flexor tendons, failure occurred predominantly in the transition of the clamping area in 29/50 samples (58%) being used for the *fatigue* testing. Of the total of 79 flexor tendons used, material wear and failure of the clamps occurred as given in Table 1 (2 clamps per tendon, 158 clamps in total, single use).

**Table 1:** Number and type of clamp failures in the *cyclic* (*fatigue*) setup for the 158 clamps used for 79 tendons. Note: One clamp showing both modes of longitudinal failure.

Failure type	Absolute number and relative %	
Transverse edge	13	8%
Transverse middle	0	0%
Longitudinal edge	47	30%
Longitudinal middle	1	1%
Total failures	60	38%
**Total Number**	**158**	

**Figure 10:**
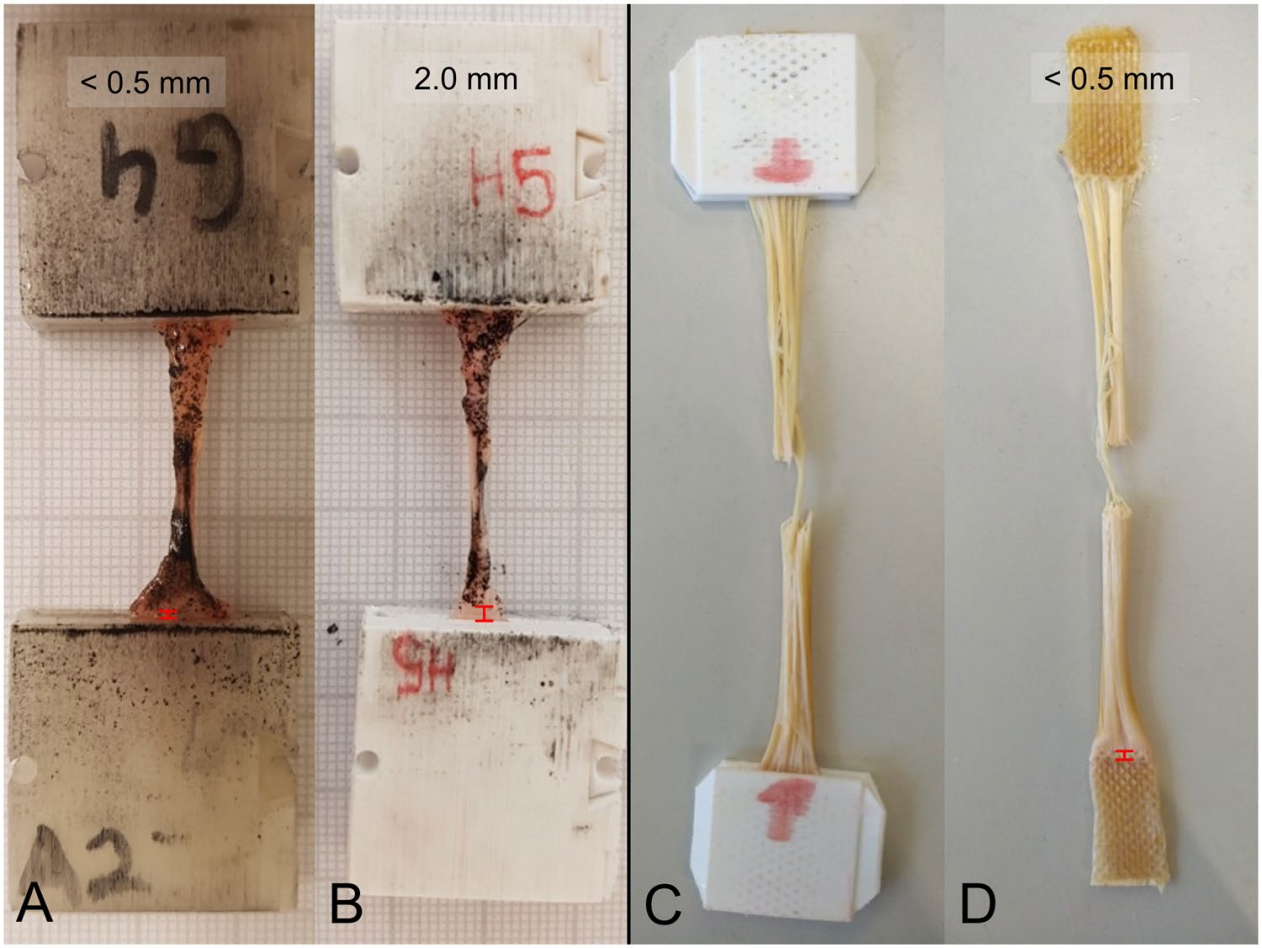
Examples of low to minute material slippage in 3D printed clamps for *quasi-static* (**A**,**B**) and *fatigue* testing (**C**,**D**) indicated by red distance signs at the lower parts of each picture.

Material slippage using the 3D printed clamps for the *quasi-static* experiments varied and was qualitatively dependent on sample thickness; yet with no statistically significant level of correlation (p > 0.05). Figure 10 shows the ligament example with the most pronounced slippage (2.0 mm) and a representative comparison with minute slippage. As these *quasi-static* experiments were synchronized with DIC data of the specimen deformation, the error in strain recording potentially resulting from the slippage could be ruled out effectively. During the *quasi-static* experiments, no clamping failure was observed. However, as a result of repeated opening and closing and in particular for thick samples (skin tests), some of the clamps showed signs of weakening and were consequently excluded from further use.

In the *fatigue* tests, material slippage was generally low, likely as a consequence of the lower stresses compared to the *quasi-static* experiments. In most samples, slippage was minimal and below the level of macroscopically-visible detection. Also, given the nature of these tests with partial failure as a consequence of repeated loading, it was impossible to separate slippage from preconditioning phenomena (stretching and parallelization of collagens). As the primary objective of the *fatigue* tests was, however, to obtain load-dependent life cycles ranging beyond 10^5^ to 10^6^; speckle-based image correlation would have been impossible as the repetitive moistening of the samples would have washed off surface marking.

### Scanning electron microscopy

Of the representative samples taken for SEM, site-immanent characteristics were found for both skin and ligament samples. In the clamping area of the skin and ligament samples, imprints of the pyramid patterns were seen, causing contact patterns in the epidermal and ligament surface regions, especially in the keratins and collagens (Figs. 2B and 7; Table 2). In higher magnifications, partial collagen damage was observed. The edge regions between the unclamped and clamped parts showed similar findings with a decreased presence of collagen bundles, which tended to be more disrupted than in the clamping area. At the failure site, the existence of keratins was minimal and frequently disrupted; collagens were present throughout this region, showing partial disruption with curling, elongation and retraction of the collagen proteins. Both existence (p ≤ 0.033) and integrity (p < 0.001) of collagens and keratins were significantly site-dependent, with a strong site association for keratin and a moderate association for collagen, indicating that both keratins and collagens are more likely to remain intact at the clamps.

**Table 2:** Evaluation from scanning electron microscopy regarding collagen and keratin existence and integrity in three different areas of human skin after testing: Clamped area, transition/edge between clamping and testing area and in the area of failure.

Extra-cellular matrix type (n = 48)	Frequency (existence)		Correlation	Extracellular matrix integrity				Correlation
•Area of sample				fully intact	partially disrupted	fully disrupted		
Collagen								
• Clamp	81%		Cramér’s	23%	54%	23%		Cramér’s
• Edge	56%		φ = 0.37	11%	56%	33%		φ = 0.40
• Failure	100%		*p* = 0.033	6%	88%	6%		*p* < 0.001
Keratin								
• Clamp	100%		Cramér’s	19%	69%	13%		Cramér’s
• Edge	100%		φ = 0.86	38%	50%	13%		φ = 0.68
• Failure	13%		*p* < 0.001	0%	0%	100%		*p* < 0.001

All specimens were tested in uniaxial *quasi-static* tensile tests until failure using 3D printed clamps made of polylactic acid with pyramid structures. Note: Percentages rounded to whole numbers.

In the ligament samples, collagens were observed at all areas, including the clamps, the edge between the clamped and unclamped regions. At the clamps, fully intact and partially disrupted collagens were predominantly found, at the edge exclusively partially ruptured collagens, and at the failure site exclusively fully ruptured collagens (Table 3). Significant correlations were found for the different areas and collagen integrity, indicating that collagens are more likely to remain intact in the clamping area and consequently less damaged as a consequence of the clamping (p = 0.003). Figure 8 summarizes the ultrastructural findings from SEM for the three different areas of the clamp.

**Table 3:** Evaluation from scanning electron microscopy regarding collagen existence and integrity in three different areas of human ligaments after testing: Clamped area, transition/edge between clamping and testing area and in the area of failure.

Extra-cellular matrix type (n = 12)	Frequency (existence)		Correlation	Extracellular matrix integrity				Correlation
•Area of sample				fully intact	partially disrupted	fully disrupted		
Collagen								
• Clamp	100%		n/a	50%	50%	0%		Cramér’s
• Edge	100%			0%	100%	0%		φ = 0.816
• Failure	100%		n/a	0%	0%	100%		p = 0.003

All specimens were tested in uniaxial *quasi-static* tensile tests until failure using 3D printed clamps made of polylactic acid with pyramid structures.

### Assessment of sterilization-induced deformations

**Figure 11:**
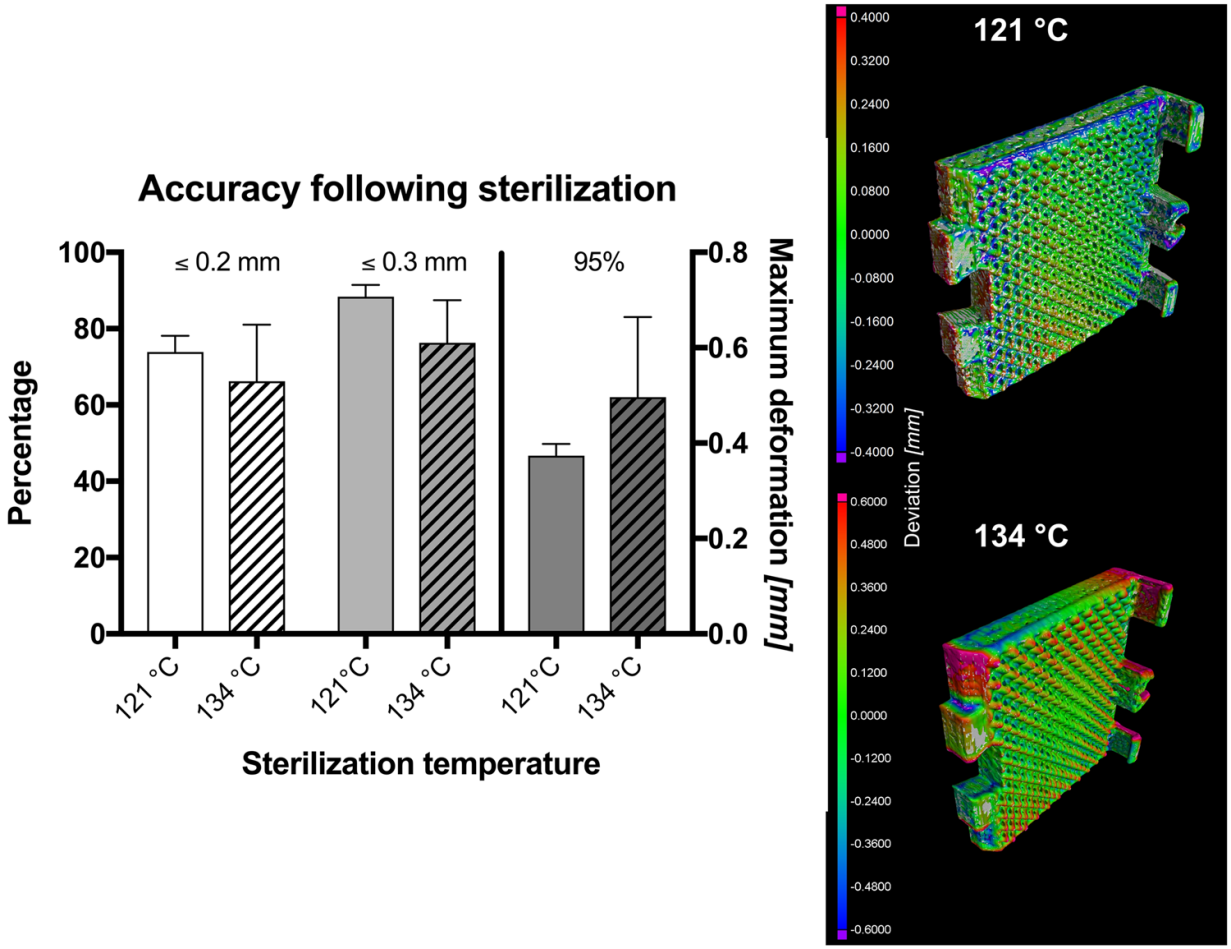
Graph summarizing the accuracy of the 3D printed clamps following sterilization using two different protocols. Percentage indicates the amount surface identical prior to and following sterilization within the given range of accuracy. The 95% value indicates the maximum deviation observed for 95% of the clamp surface. The illustrated an overlay between the initial shape and the shape of the clamps following sterilization.

Following the sterilization, the optically visible deformation was minute. However, quantifiable deformations were observed by μCT for the clamps at both temperatures. Using the 121 °C protocol, 73.9 ± 3.5% of the clamp surface matched with the original shape within 0.2 mm deviation, and 88.34 ± 2.5% within 0.3 mm. These values were similar to the 134 °C protocol, where the surfaced of the sterilized clamps matched with the original shape in 66.2 ± 12.1% within 0.2 mm and 76.3 ± 9.1% within 0.3 mm, respectively. Using the 121 °C protocol, 95% of the surface varied less than 0.38 ± 0.02 mm, and 0.50 ± 0.14 mm for the 134 °C protocol. The deformations observed are summarized in Fig. 11 and were located especially at the margins and edges of the clamps, not allowing to continue using the clamps reliably including the locking mechanism.

## Discussion

In summary, 3D printing of clamps and fixtures provides a number of advantages over other techniques, making it particularly suitable if a relatively high throughput is required under a standardized testing environment for investigation of sufficient high numbers of test samples in biomedical research. It moreover, in *quasi-static* testing, allows for pre-determining the area of failure in a central specimen region, as indicated by SEM. This feature is of particular importance if one may hypothesize that failure might primarily occur at the clamps due to avulsion. For *fatigue* tests, the given design will need further refinement if a similar pre-determination of the rupture area is desired or necessary. This may potentially involve tapering of these samples, likely with negative impact on the number of load cycles to failure.

A variety of soft tissue clamping methods exist, which are often adapted from engineering materials such as plastic, carbon or glass fiber composites. As an example, European DIN EN ISO 527 T5 (combined with probe geometries according to the DIN EN ISO 527 T4) allows for sufficient testing with fiber orientations deviating up to 45 degrees from the direction of load application but uses strong adhesives to mount the probe to the fixture. It is however unclear to which extent the chemicals and solvents in the adhesives will alter the areas of the clamped tissues, and this may also impact on the areas outside of the clamping. This applies also to the partial plastination technique^15,16^. Roughened steel or sand paper-reinforced clamps form further soft tissue clamping alternatives^1–7^ but have disadvantages regarding the repeatability of the mounting procedure and the re-use of the clamps themselves. To overcome material slippage, a number of adjustments and fixtures have been introduced^1–3,6–9^. Cryogenic clamping brings about further temperature (or freeze breaking) induced changes in material properties. Other adjustments in clamping have attempted to address the issue of stress concentrations at standard friction clamps, e.g. roller fixtures^22^. Such adjustments however have the disadvantage that a relatively long sample length is required, which is usually difficult to achieve with most soft tissues, especially when testing human samples. Moreover, bone-ligament-bone complexes^13,23^ are difficult to be obtained and standardized especially if samples are obtained as part of a biopsy only, and they do not exclusively reflect the mechanical properties of the ligament of interest. As shown in this given study, the clamping using 3D printed PLA clamps appears not to have a negative influence on the integrity of the extracellular matrix in the testing area and at the transition to the clamps to the end that the clamping is a major source of material failure.

The given method with 3D printing therefore addressed successfully the primary aim at improving standardization, while simultaneously allowing for high-volume sample testing in biomechanical setups. The method has rendered reliable results for human soft tissues, though further refinements will be necessary depending on the nature of the proteins composing the samples and the type of experiment running. Table 4 summarizes in brief the features of sand paper clamping, partial plastination, cryogenic clamping and 3D printed clamps as comparison between well-known and new clamping techniques. It can be summarized that the given clamping design adds a novel tool for specimen mounting to the field of soft tissue biomechanics, providing a standardized and easy to manufacture clamping method that is useful for a variety of adjustments in customized tests.

**Table 4:** Comparison of four clamping techniques and their suitability for biomechanical testing.

	Sand paper clamping	Partial plastination	Cryogenic clamping	3D printed clamping system
Level of standardization (e.g. specimen alignment, clamping length)	Low	Moderate	Moderate	High
Ease of use	Easy	Complex	Moderate	Easy
Use of potentially dangerous goods?	No	Yes (solvents & resin)	Yes (liquid N_2_)	No
Adaptability to various tissues	None	None	Moderate	High
Modularity	None	None	None	High
Material slippage	High	Minute	Low	Low
Work time involved for preparation excluding testing	15 mins/sample	30 mins/sample	15 mins/sample	10–15 mins/sample
Applicability for *quasi-static* testing	Suitable	Suitable	Suitable	Suitable
Applicability for *cyclic* and *fatigue* testing	Unsuitable (material slippage)	Unsuitable (avulsion at the clamping interface)	Unsuitable (continuous cooling necessary, temperature decrease of tissues)	Suitable for ≥10^5^ cycles
Throughput	high	low	moderate	high
Type & price of consumables and mounting materials per specimen	Sandpaper ca. 0.30 NZ$ (0.18 US$/€)	Acetone, resin 5–7 NZ$ (3–4 US$/€)	Liquid nitrogen 1 NZ$/l (0.60 US$/l)	Printing materials ca. 0.50 NZ$ (0.30 US$/€)
Reusability of consumables	not given	not given	not given	estimated 3–5×*
Desinfectability	poor	poor	good	good
Sterilizability	poor	poor	good	poor
Suitable field of application	Preliminary tests	Highly-specialized applications	Small sample sizes, high tensile forces	High-standardization-high-throughput experiments

^*^Applied to aseptic conditions using 50% ethanol or Viraclean^®^.

The key advantages of the 3D printed clamps, supporting arms, tables and templates are the ease of use, adaptability, reusability, modularity and the fact that they can be manufactured easily and at local research environments. Furthermore, recent developments in prices, usability and variety of desktop FDM-printers (which was used in this study) allow an easy integration into laboratory environments. Without the maintenance time of the printer, which is often reduced to some minutes (when changing a filament spool or cleaning the build-plate), no additional working time is required for the manufacturing of the parts for testing. Conventional 3D printing materials like PLA and TPU can be printed easily without any hazard of air contamination and are available as single filaments spools for moderate prices (e.g. PLA: from 30 NZ$/kg), which can be used for printing e.g. a couple of hundred clamps. Geometries and print settings can be shared easily, forming a basis for affordable add-ons to existing testing devices for tissue biomechanics. Given that no specialized equipment or procedures are required for the preparation of the samples, the entire pre-testing procedure can be conducted immediately before to the actual experiments in the testing environment.

A limitation of the 3D printing techniques utilizing PLA or TPU is formed by the relatively low melting temperatures, which limit the possibility of PLA- and TPU-based plastics to be sterilized. This is especially the case with autoclaving or dry heat as the widest spread sterilization techniques^24,25^, which has been confirmed by our results in μCT based visualization. Ethylene oxide and gamma irradiation may provide a solution here^24,25^. However, it needs to be acknowledged that for most testing conditions, aseptic conditions provide a sufficient environment and that a sterile environment is difficult to be achieved with most mechanical testing machines. If sterile conditions must apply, customized clamps printed of heat and chemically resistant metal powders or milled fixtures may be preferential. Furthermore, when using metals, it is unclear if the pyramid structures or the snapping mechanism may then need further adjustments to minimize avulsion between the sharp geometries.
